# Enhancing Quality of Life in the Elderly: The Impact of Psychosomatic Exercises on Healthy Aging

**DOI:** 10.1192/j.eurpsy.2024.654

**Published:** 2024-08-27

**Authors:** K. Argyropoulos, R. Sfakianaki, A. Argyropoulou, D. Avramidis, P. Gourzis, E. Jelastopulu

**Affiliations:** ^1^Public Health, School of Medicine; ^2^Social Sciences, Hellenic Open University; ^3^ Health Center of Andravida; ^4^School of Medicine, Patras, Greece; ^5^Psychiatry, School of Medicine, Patras, Greece

## Abstract

**Introduction:**

Older individuals constitute a significant portion of the population, and concerted efforts are underway to enhance the quality of this life stage by minimizing health issues and maximizing opportunities.

**Objectives:**

This study aims to investigate the impact of psychosomatic exercises, including practices like yoga, meditation, and tai chi, as an alternative approach to promoting healthy aging and ultimately enhancing the quality of life among elderly individuals.

**Methods:**

The study comprised 84 participants, with 51 individuals engaging in various forms of psychosomatic exercises and 33 serving as the control group, having no prior exposure to such practices. Data collection was carried out electronically, with the initial section gathering socio-demographic information and health-related details about the participants. The second part consisted of the WHOQOL-BREF quality of life scale, consisting of 26 questions, which assessed six domains: Overall Quality of Life and General Health, Physical Health, Psychological Health, Social Relationships, and Environment. Statistical analysis was performed with SPSS 26.

**Results:**

The average age of the participants was 66.7 years. A statistically significant positive correlation was identified within the first subscale of the tool, “Overall Quality of Life and General Health,” with scores of 74.3/100 for those engaging in psychosomatic exercises and 66.7/100 for those who did not (t(82) = -2.513, p = 0.014). However, no statistically significant differences were observed in the remaining subscales.

**Conclusions:**

Psychosomatic exercises, including yoga, meditation, and tai chi, hold promise as a means to improve the overall quality of life and general health of elderly individuals. These practices could serve as valuable components of strategies aimed at promoting healthy aging. Further research is needed to explore their effects in greater detail and across various dimensions of well-being.

**Disclosure of Interest:**

None Declared

